# Non‐Cardiogenic Pulmonary Oedema Provoked by Acetazolamide

**DOI:** 10.1002/rcr2.70118

**Published:** 2025-02-11

**Authors:** Naser Naser, Salma Shehabi, Khaled Maki

**Affiliations:** ^1^ The Royal College of Surgeons in Ireland of Bahrain (RCSI Bahrain) Busaiteen Bahrain

**Keywords:** acetazolamide, intraocular pressure (IOP), non‐cardiogenic pulmonary oedema (NCPE)

## Abstract

A 61‐year‐old male was brought to the Emergency Department with severe shortness of breath, a throbbing headache, sweating, nausea, vomiting and diarrhoea after the administration of an acetazolamide tablet (250 mg) at a private ophthalmology clinic. On presentation, a chest X‐ray was performed, showing diffuse alveolar opacities bilaterally, indicating pulmonary oedema, as seen in CT chest also. However, his echocardiogram revealed a normal ejection fraction with no signs of ischemia. He was subsequently diagnosed with non‐cardiogenic pulmonary oedema (NCPE) and was immediately started on high‐flow oxygen, later requiring mechanical ventilation. The patient was admitted to the Critical Care Unit with supportive treatment, including IV fluids and antibiotics, without steroid administration. Four days later, he was extubated and subsequently discharged from the ICU, followed by discharge from the hospital. Our case revolves around a rare yet potentially fatal episode of NCPE secondary to Acetazolamide use.

## Graphical abstract

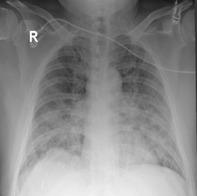

## 1 Introduction

Acetazolamide is a medication that is often used by ophthalmologists to reduce the intraocular pressure (IOP) of the eye [1]. It inhibits an enzyme called carbonic anhydrase in the ciliary processes, thus reducing aqueous flow and resulting in reduced IOP. This is a case report of a 61‐year‐old male who was admitted to the Intensive Care Unit with acute Non‐Cardiogenic Pulmonary Oedema (NCPE) secondary to Acetazolamide.

## 2 Case Report

A 61‐year‐old male was brought to the Emergency Department with severe shortness of breath, a throbbing headache, sweating, nausea, vomiting and diarrhoea after the administration of an acetazolamide tablet (250 mg) at a private ophthalmology clinic.

In December 2018, the patient had an appointment at the ophthalmology clinic for treatment of a macular hole in his eye. He was given an acetazolamide tablet half an hour before the procedure. After the intervention was completed, a follow‐up appointment was scheduled.

A month later, the patient arrived at the clinic and was given an acetazolamide tablet once again. Roughly 20–25 min later, he began experiencing severe shortness of breath, described ‘feeling very hot’, developed a throbbing headache, and had nausea associated with four episodes of vomiting and three episodes of diarrhoea. This was the patient's first presentation of such symptoms. Notably, there was no history of flu‐like symptoms, cough or fever prior to presentation.

**FIGURE 1:**
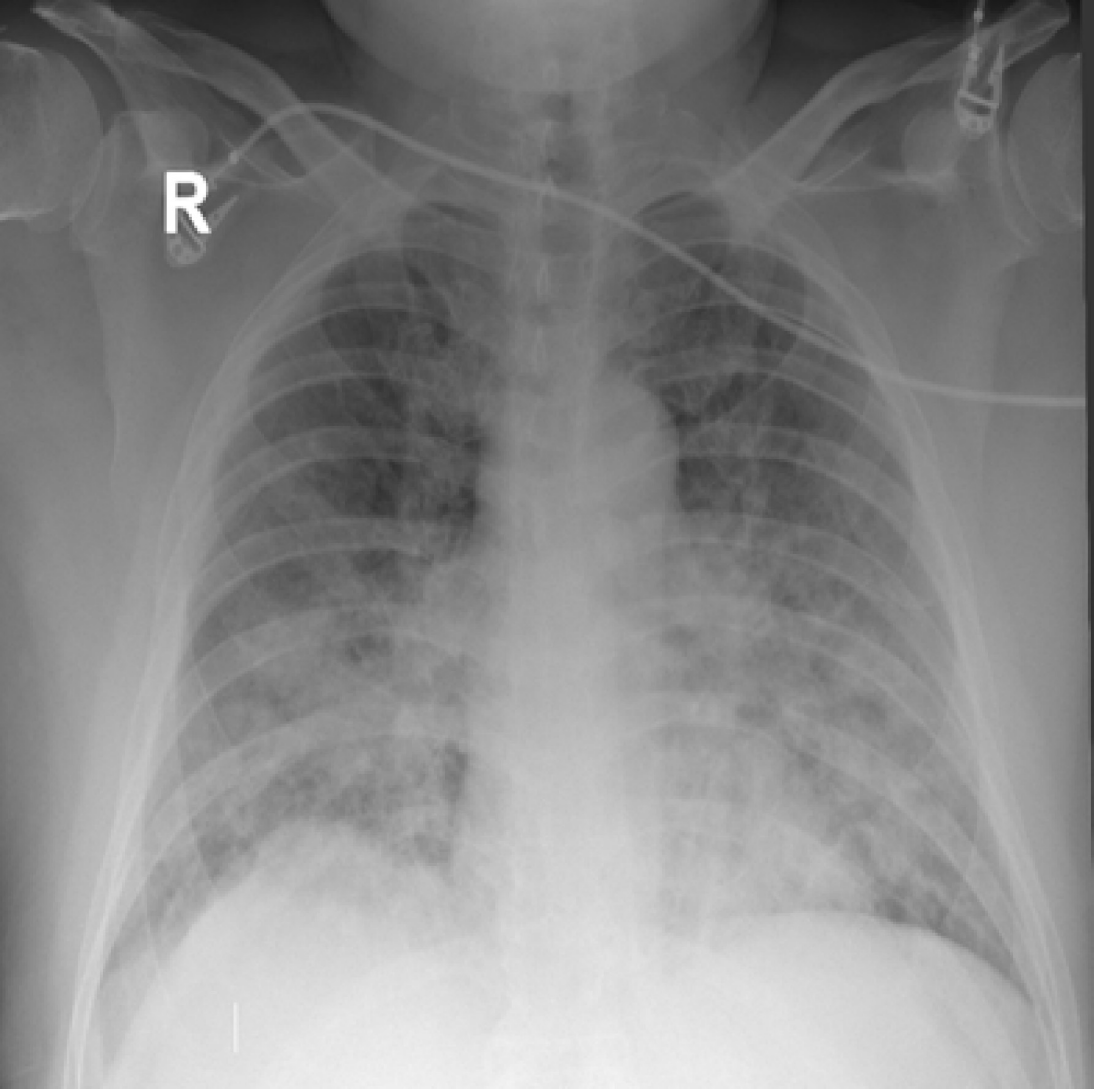
Chest X‐ray at presentation showing diffuse bilateral alveolar shadowing.

**FIGURE 2:**
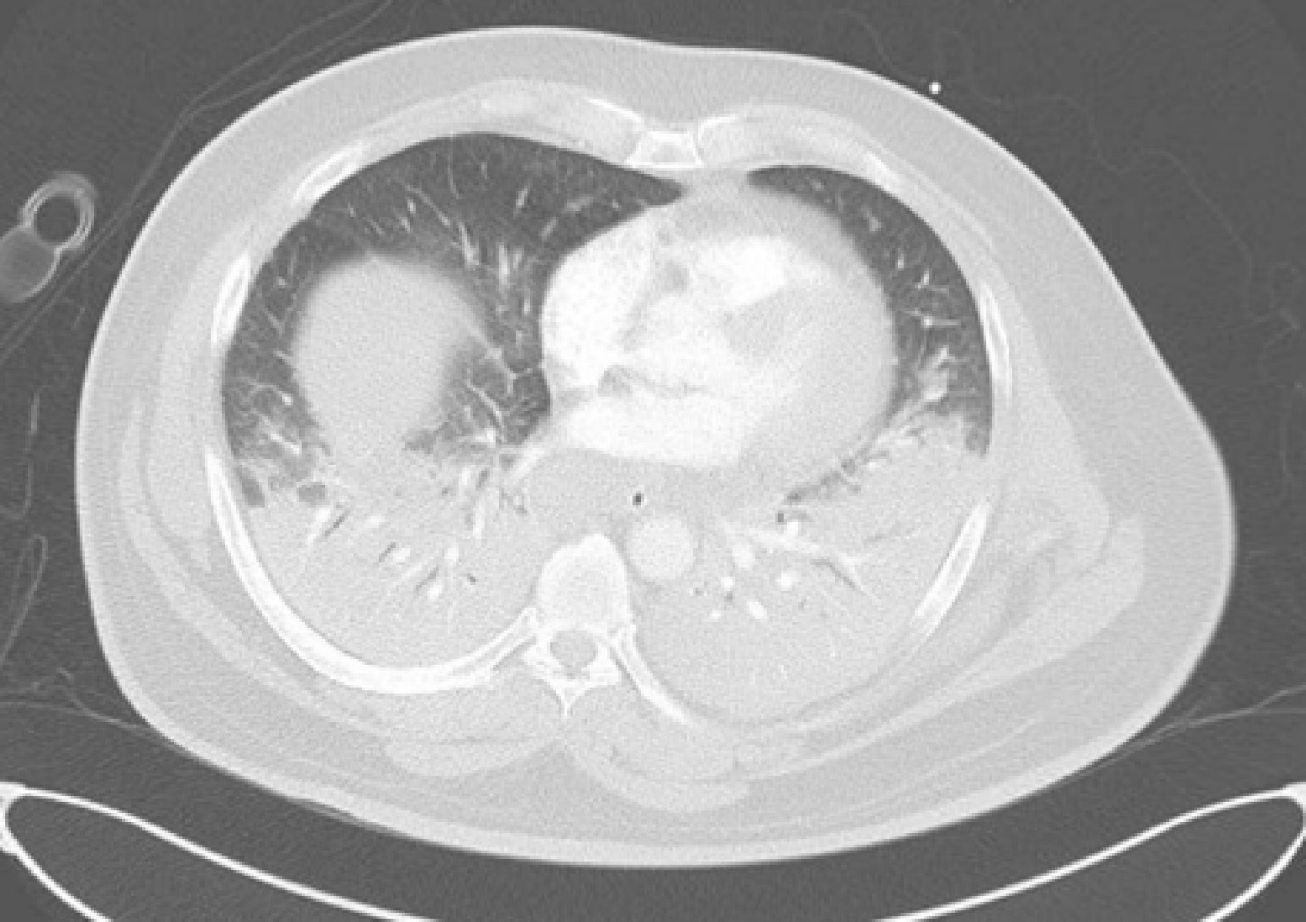
CT chest showing bilateral diffuse consolidation without pleural effusion.

On presentation, a chest X‐ray (Figure 1) was performed, showing diffuse alveolar opacities bilaterally, indicating pulmonary oedema as seen in CT chest also (Figure 2). However, his echocardiogram revealed a normal ejection fraction with no signs of ischemia. He was subsequently diagnosed with NCPE and was immediately started on high‐flow oxygen, later requiring mechanical ventilation.

His initial laboratory results showed a normal full blood count and an ESR of 8 mm/h, with elevated procalcitonin levels. Acute kidney injury was noted, likely due to circulatory collapse from the drug reaction and over‐diuresis prior to admission.

The patient was admitted to the Critical Care Unit with supportive treatment, including IV fluids and antibiotics, without steroid administration. All cultures, including blood, sputum, and urine, were sterile. PCR tests for Influenza and Respiratory Syncytial Virus (RSV) were also negative.

Four days later, he was extubated and subsequently discharged from the ICU, followed by discharge from the hospital.

## 3 Discussion

Carbonic anhydrase inhibitor (CAI) drugs, whether used topically or systemically, are generally considered safe [2]. However, NCPE due to Acetazolamide is an extremely rare adverse effect, which may occur after the first dose or with subsequent doses [3].

Our patient, generally healthy, attended a routine follow‐up appointment during which he received oral Acetazolamide. During his hospital admission, he exhibited a significantly elevated procalcitonin level (> 100) but showed no signs of active infection or pneumonia. This suggests that procalcitonin may not be a reliable marker for distinguishing between drug‐related and sepsis‐related acute respiratory distress syndrome (ARDS). Despite a history of safe Acetazolamide use a month ago, the patient developed non‐cardiogenic pulmonary oedema.

The exact mechanism behind Acetazolamide‐induced NCPE is not well understood. It is likely an idiosyncratic reaction involving capillary leakage, anaphylaxis, or drug‐induced hypervolemia. Previous case reports [4] indicate that symptoms typically appear within 1 h and may include nausea, vomiting and dyspnoea, sometimes accompanied by fever and hypotension.

NCPE can occur with both oral and intravenous Acetazolamide administration. Recovery from Acetazolamide‐induced NCPE can range from hours to days; however, the condition can be fatal. Although distinguishing drug‐induced from infection‐induced NCPE can be challenging, in our case, the clinical presentation was clearly linked to Acetazolamide administration.

Acetazolamide‐induced NCPE is usually managed [2, 3, 4, 5] with supportive measures, including oxygen and mechanical ventilation in severe cases (either invasive or non‐invasive). The most critical challenge in management is excluding other causes of pulmonary oedema. If confirmed as Acetazolamide‐induced NCPE, it is essential to discontinue the drug and avoid future rechallenges, as this could lead to fatal outcomes. The role of steroids and diuretics remains controversial, as over‐diuresis can sometimes result from misdiagnosis.

Our case was managed solely with ventilatory support and intravenous fluids to address acute kidney injury (AKI) resulting from NCPE and initial overuse of diuretics.

In conclusion, our case revolves around a rare yet potentially fatal episode of NCPE secondary to Acetazolamide use. With attention to both our experience with this patient's case and to several case reports that have discussed similar incidents, we believe that more light should be shed on Acetazolamide‐triggered NCPE. Moreover, further research is required to understand the mechanisms of this condition and the groups of patients at higher risk of developing this adverse effect.

## Author Contributions

S.S. and K.M. drafted the manuscript. N.N. participated in the literature search and edited the final manuscript. All authors read and approved the final manuscript.

## Ethics Statement

The authors declare that appropriate written informed consent was obtained for the publication of this manuscript and accompanying images.

## Conflicts of Interest

The authors declare no conflicts of interest.

## Data Availability

The data that support the findings of this study are available on request from the corresponding author. The data are not publicly available due to privacy or ethical restrictions.

## References

[1] American Academy of Ophthalmology , Acetazolamide: Considerations for Systemic Administration [Internet] (American Academy of Ophthalmology, 2015), https://www.aao.org/eyenet/article/acetazolamide‐considerations‐systemic‐administration.

[2] M. M. Popovic , M. B. Schlenker , D. Thiruchelvam , and D. A. Redelmeier , “Serious Adverse Events of Oral and Topical Carbonic Anhydrase Inhibitors,” JAMA Ophthalmology 140, no. 3 (2022): 235–242, 10.1001/jamaophthalmol.2021.597. 35084437 PMC8796060

[3] I. Vogiatzis , E. Koulouris , A. Sidiropoulos , and C. Giannakoulas , “Acute Pulmonary Edema After a Single Oral Dose of Acetazolamide,” Hippokratia 17, no. 2 (2013): 177–179. 24376328 PMC3743627

[4] R. Prasad , P. Gupta , A. Singh , and N. Goel , “Drug Induced Pulmonary Parenchymal Disease,” Drug Discoveries & Therapeutics 8 (2014): 232–237. 25639301 10.5582/ddt.2014.01046

[5] A. H. Schwartz and S. Sieminski , “Acetazolamide Induced Noncardiogenic Pulmonary Edema, an Underreported Serious Adverse Event,” American Journal of Ophthalmology Case Reports 30 (2023): 101827, 10.1016/j.ajoc.2023.101827. 37034462 PMC10074496

